# Germs of thrones - spontaneous decolonization of Carbapenem-Resistant Enterobacteriaceae (CRE) and Vancomycin-Resistant Enterococci (VRE) in Western Europe: is this myth or reality?

**DOI:** 10.1186/s13756-018-0390-5

**Published:** 2018-08-13

**Authors:** Benjamin Davido, Aurore Moussiegt, Aurélien Dinh, Frédérique Bouchand, Morgan Matt, Olivia Senard, Laurene Deconinck, Florence Espinasse, Christine Lawrence, Nicolas Fortineau, Azzam Saleh-Mghir, Silvia Caballero, Lelia Escaut, Jérome Salomon

**Affiliations:** 1 Maladies infectieuses, Hôpital Universitaire Raymond-Poincaré, AP-HP, 92380 Garches, France; 2 Pharmacie Hospitalière, Hôpital Universitaire Raymond-Poincaré, AP-HP, 92380 Garches, France; 3 Hygiène Hospitalière, Hôpital Universitaire Ambroise-Paré, AP-HP, 92210 Boulogne-Billancourt, France; 4 Hygiène Hospitalière, Hôpital Universitaire Raymond-Poincaré, AP-HP, 92380 Garches, France; 5 Laboratoire de Microbiologie, Hôpital Universitaire Kremlin-Bicêtre, AP-HP, 94270 Le Kremlin-Bicêtre, France; 6 Vedanta Biosciences Inc, Cambridge, MA 02139 USA; 7 Maladies Infectieuses, Hôpital Universitaire Kremlin-Bicêtre, AP-HP, 94270 Le Kremlin-Bicêtre, France

**Keywords:** Decolonization, Carbapenem-Resistant Enterobacteriaceae, Vancomycin-Resistant Enterococcus

## Abstract

**Background:**

In France, Carbapenem-Resistant Enterobacteriaceae (CRE) and Vancomycin-Resistant Enterococci (VRE) are considered as Extensively Drug-Resistant (XDR) bacteria. Their management requires reinforcement of hospital’s hygiene policies, and currently there is few consistent data concerning the spontaneous decolonization in XDR colonized patients. Our aim is to study the natural history of decolonization of XDR carriers over time in a hospital setting in a low prevalence country.

**Material and methods:**

Retrospective multicenter study over 2 years (2015–2016) in 2 different tertiary care hospital sites and units having an agreement for permanent cohorting of such XDR carriers. We gathered the type of microorganisms, risk factors for colonization and rectal swabs from patient’s follow-up. We also evaluated patient care considering isolation precautions.

**Results:**

We included 125 patients, aged 63+/−19y, including 72.8% of CRE (*n* = 91), 24.8% of VRE (*n* = 31) and 2.4% (*n* = 3) co-colonized with CRE and VRE. CRE were mainly *E. coli* (*n* = 54), *K. pneumoniae* (*n* = 51) and *E. cloacae* (*n* = 6). Mechanisms of resistance were mainly OXA-48 (*n* = 69), NDM-1 (*n* = 11), OXA-232 (*n* = 8) and KPC (*n* = 3).

Prior antibiotic therapy was reported in 38.4% (*n* = 48) of cases. Conversely, 17.6% (*n* = 22) received antibiotics during follow-up.

Spontaneous decolonization occurred within the first 30 days in 16.4% (*n* = 19/116) of cases and up to 48.2% after day-90 with a median follow-up of 96 days (0–974).

We estimated that XDR carriage was associated with a larger care burden in 13.6% (*n* = 17) of cases, especially due to a prolongation of hospitalization of 32.5 days (15–300).

**Conclusions:**

Our study shows that spontaneous decolonization is increasing over time (up to 48.2%). We can regret that only few patients underwent screening after 1 year, emphasizing the need for more monitoring and prospective studies.

## Introduction

In recent years, multidrug-resistant organisms (MDRO), especially Vancomycin-Resistant Enterococcus (VRE) and Carbapenem-Resistant Enterobacteriaceae (CRE) [1] are becoming more prevalent worldwide and represent a serious public health threat since they are becoming increasingly resistant to current treatment drugs. Moreover, CRE infections have been associated with an increased risk of mortality in Europe [2, 3].

In France, VRE and CRE belong to a group of Extensively Drug-Resistant (XDR) bacteria. They are spreading worldwide and oblige us to reinforce our in-hospital hygiene policy [4]. It has been suggested by the CDC [5] and national guidelines [6] that patients carrying such microorganisms must be ideally placed in isolation in a specific ward and cared for by a dedicated paramedical team, notably nurses. Nonetheless, it is well known that healthcare workers spent less time with patients in isolation which impacts negatively patient mental well-being [7].

Furthermore, no specific treatment is currently available to promote decolonization of these MDROs. Indeed, decolonization strategies have resulted in colonization relapse with the emergence of resistance to the intended therapies [8–10]. More recently, fecal microbiota transplantation (FMT) has been proposed as a promising treatment option to clear XDR carriage in mice [11] and in humans [12–14], including immunocompromised individuals [15]. To date, there are no randomized clinical trial data to support its efficacy, but previous cited findings suggest that FMT might be more effective against VRE than CRE colonization. Yet, as FMT in such indication is still under evaluation it cannot be performed in common clinical practice, thereby leaving physicians without immediate solution.

A recent systematic review by Bar-Yoseph et al. showed that in the natural history of colonization among healthcare residents, a significant proportion of CRE carriers (34.6%) remained colonized for up to 1 year [16]. While this meta-analysis is of interest, it is mainly composed of 4 cohorts which benefited simultaneously from active decolonization therapy and thus did not focus on spontaneous decolonization.

As data are limited in the literature, we decided to analyze the natural history of spontaneous decolonization in XDR colonized patients and identify whether XDR carriage was associated with a larger care burden with a difference from the standard patient care because of the cohorting strategy recommended in acute healthcare facilities [5].

## Methods

### Study setting

The hospital group Paris-Ile-de-France Ouest is composed of 2 main sites known as Raymond-Poincaré hospital and Ambroise-Paré hospital. Both are tertiary care hospitals with acute care facilities (255 and 750 beds, respectively) and share the same emergency unit with approximately 28,400 admissions per year for complete hospitalization.

Kremlin-Bicêtre hospital is a tertiary care hospital with acute care facilities [1027 beds, including 55 beds in the adult intensive care unit (ICU)] with also medium and long-term care facilities (230 beds). There are ca. 20,000 admissions per year for complete hospitalization from emergency unit.

These 2 hospitals are the only ones in the Paris area to have an agreement for permanently dedicated staff and beds to hospitalize patients colonized with XDR.

Raymond-Poincaré hospital unit of Infectious Disease can accommodate up to 31 patients, including 5 to 8 beds in a dedicated unit for the management of XDR carriers, whereas Kremlin-Bicêtre hospital has a 26-bed infectious diseases unit with 6 beds dedicated to the XDR carriers.

Patients were systematically screened every week with rectal swabs sent to the operational hygiene team for monitoring and follow-up.

We performed a retrospective multicenter study and included all patients hospitalized in these specific units with previously known or confirmed XDR colonization.

### Data collection

Data were collected from January 2015–December 2016 in both dedicated units.

As a first step, medical charts were reviewed through computerized charts including clinical and laboratory data.

The following data were recorded:

- patient characteristics: past medical history, age, sex, ethnic origin; Charlson Score Index (CCI);
- ideal hospital unit required for patient care and length of stay (LOS);
- type of MDRO and their mechanism of resistance;
- possible cause of colonization and risk factors associated with it;
- ongoing or previous antimicrobial therapy: international drug name and duration; and
- follow-up swabs to assess decolonization (median time and duration over time). We considered decolonization as a clearance after at least 2 negative specimens with at least a one-week interval

Consecutively, 2 independent infectious disease specialists (IDS) determined whether patients experienced a disrupted medical care, by taking into account all the cited above data. A larger care burden was defined by a prolonged LOS and a delay in their management according to the main diagnosis of hospitalization in comparison to the average LOS obtained through the Computerized Medical Information Systems Program (MISP), or in their hospital discharge (i.e. a patient who requires surgery and is not operated because of a MDRO carriage).

### Microbiological data

Swabs were performed using sterile transport swab (ref. 139C) on specific media (liquid Stuart, Venturi Transystem*®* by COPAN Italia, Brescia, Italy).

Each swab was cultivated on specific selective media (chromID*®* VRE and chromID® CARBA SMART by bioMérieux, Marcy l’Etoile, France), and on regular MDRO agar plates according to manufacturer. No enrichment cultures were used.

Moreover, at admission colonization was systematically confirmed by PCR testing (PCR Xpert CarbaR and Xpert VanA/VanB by Cepheid, Maurens-Scopont®, France) and during follow-up in case of negative culture on the demand of physician.

### Statistical analysis

Statistical analyses were performed to compare rates and study potential factors of decolonization between colonized and decolonized patients. Student’s *t*-test was performed to analyze continuous data using GraphPad Prism v.7.0 (GraphPad Software Inc., La Jolla, CA). Statistical significance was defined as *P* < 0.05.

## Results

### Epidemiological data

A total of 198 stays regarding 125 patients with a mean ± standard deviation age of 63 ± 19 years were analyzed. Sex ratio (M/F) was 2.6. Median Charlson score was 4 (IQR 3–6). Patients were colonizers in 95.2% (*n* = 119) of cases whereas we observed 6 patients infected by a XDR bacteria.

**Fig. 1:**
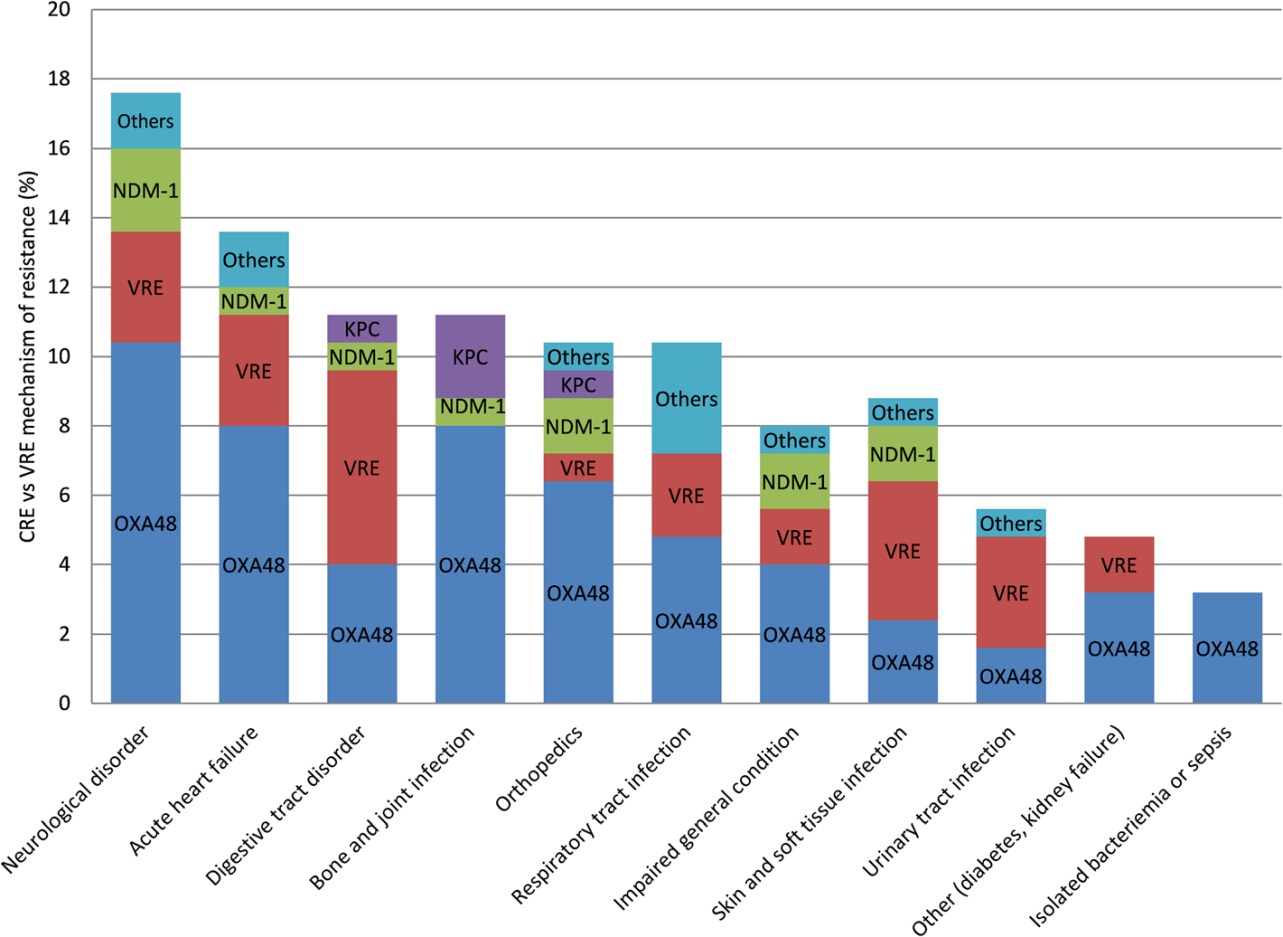
Etiologies of hospitalization depending on types of XDR carriage

Initial causes of hospitalization are summarized in Fig. 1. Infections accounted for 38.4% (*n* = 48) of etiologies on the basis of the presence of fever with a microbiological finding (such as respiratory multiplex PCR, urinalysis, blood culture, bone and deep wound specimens or stool sample). Median LOS was 12 days (range 1–290).

Considering patients who were known to be colonized before hospitalization (*n* = 31), prior median duration for colonization was 64 days (3–609). Conversely, for patients discovered colonized at admission (*n* = 94), median time for the diagnosis of colonization was 2 days (0–127) after admission.

MDRO colonization was divided as follows: 72.8% of CRE (*n* = 91), 24.8% of VRE (*n* = 31) and 2.4% of co-colonization VRE/CRE (*n* = 3).

For CRE, they were mainly composed of *E. coli* (*n* = 54) (including 21 co-colonization), *K. pneumoniae* (*n* = 51) (including 20 co-colonization), *E. cloacae* (*n* = 6) (3 co-colonization), *C. freundii* (*n* = 6) (4 co-colonization) and *E. aerogenes* (*n* = 3 co-colonization). Mechanisms of resistance were OXA-48 (75.8%; *n* = 69), NDM-1 (12.1%; *n* = 11), OXA-232 (8.8%; *n* = 8), VIM (3.3%; *n* = 3) or KPC (3.3%; *n* = 3) and NDM-5 (*n* = 1); including OXA-48/NDM-1 isolates (4.4%; *n* = 4).

For VRE, mechanisms of resistance were exclusively Van-A phenotypes.

**Fig. 2:**
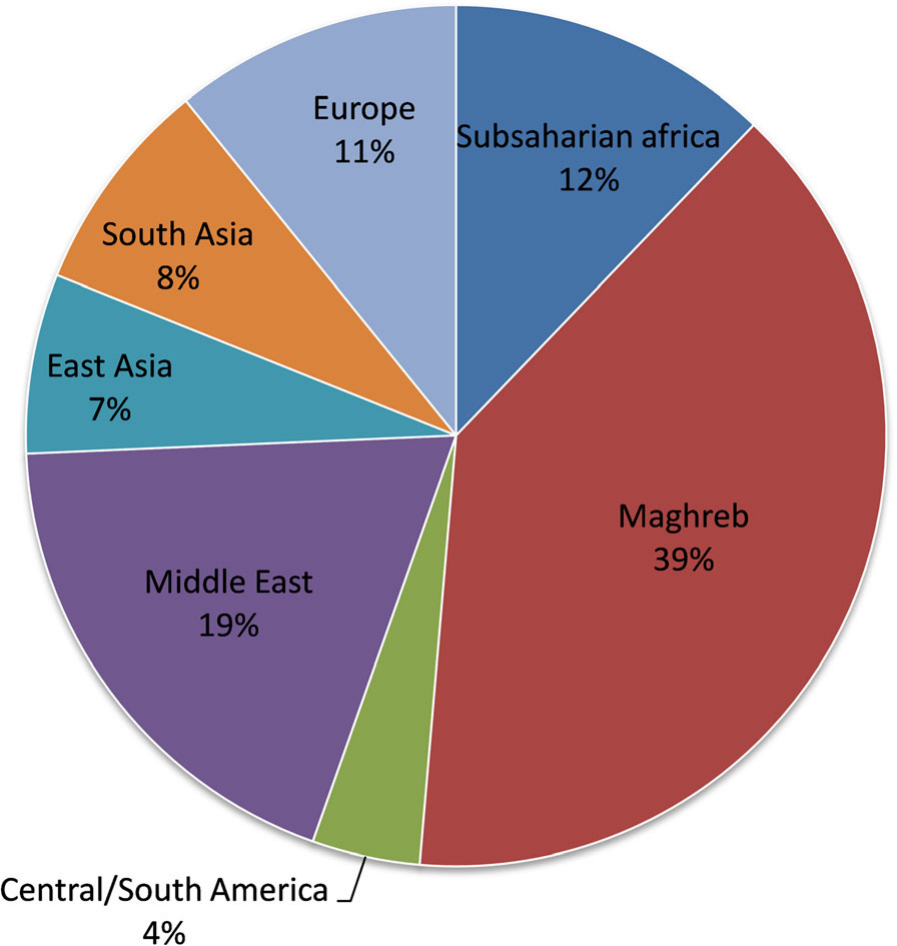
Schematic of the destination abroad within the year before hospitalization and suspected to be related to their XDR colonization (*n* = 74)

Common risk factors for the acquisition of XDR bacteria were: i) a previous trip abroad in an endemic zone within the year (59.2%; *n* = 74), including 51 local hospitalizations; ii) an in-hospital contact with an already colonized patient (23.2%; *n* = 29). Destinations considered as potentially responsible for the acquisition of resistance are illustrated in Fig. 2.

Prior antimicrobial therapy was reported in 38.4% (*n* = 48) of cases. Conversely, 17.6% (*n* = 22) received antibiotics during follow-up. Before and after regimens (*n* = 90) were mainly composed of beta-lactams (*n* = 78) and fluoroquinolones (*n* = 23). Median duration of treatment was 14 days (3–60). Antimicrobial agents are detailed in Tables 1 and 2.

**Table 1:** Antibiotic regimen received by our patients (*n* = 125), before and after admission

	N (%)
Patients with no antibiotic treatment	55 (44.0)
Patients receiving at least one antibiotic regimen	70 (56.0)
1 regimen	50 (40.0)
2 regimens	18 (14.4)
3 regimens or more	2 (1.6)
Total number of antibiotic regimen prescribed	90

**Table 2:** Types of antibiotic regimen (*n* = 90) divided into monotherapy and combination therapy

Antibiotic (International drug name)	N (%)
Monotherapy	
Amoxicillin-clavulanate	20
Piperacillin-tazobactam	9
Third-generation cephalosporin	8
Carbapenem	6
Fluoroquinolone	5
Amoxicillin	4
Glycopeptide	1
Other	2
TOTAL	55 (61.1)
Combination therapy with 2 antibiotics	
Piperacillin-tazobactam + fluoroquinolone	6
Third-generation cephalosporin + other antibiotic	6
Amoxicillin-clavulanate + Fluoroquinolone	4
Third-generation cephalosporin + fluoroquinolone	3
Carbapenem + other antibiotic	3
Amoxicillin-clavulanate + other antibiotic	3
Piperacillin-tazobactam + tigecyclin	1
Piperacillin-tazobactam + antibiotic active against MRSA^a^	2
Fluoroquinolone + rifampin	1
TOTAL	29 (32.2)
Combination therapy with 3 or more antibiotics	
Carbapenem + fluoroquinolone + aminoglycoside	2
Pipera-tazobactam + fluoroquinolone + aminoglycoside	1
Carbapenem + colistin + tigecyclin	1
Pipera-tazobactam + fluoroquinolone + linezolide	1
Carbapenem + colistin + glycopeptide	1
TOTAL	6 (6.7)

^a^MRSA: methicillin-resistant *Staphylococcus aureus*

Overall, only 4.8% (*n* = 6) of cases were infected by XDR microorganisms. There were 4 monomicrobial infections: *E. coli* OXA-48 (*n* = 2), *K. pneumoniae* OXA-48 (*n* = 1) and *E. cloacae* NDM-1 (*n* = 1) and 2 plurimicrobials including 1 *E. coli + E. aerogenes* OXA-48 and 1 *K. pneumoniae* + *E. coli* OXA-48 like. No risk factors were identified, patients had comparable median age (67 ± 27.5 years), median CCI (6 ranging from 0 to 8), identical median LOS = 12 days and prior use of antibiotics (*n* = 2). They presented pyelonephritis (*n* = 2), bone and joint infections (*n* = 2), mediastinitis (*n* = 1) and cholecystitis on a cancerous biliary obstruction (*n* = 1).

### Follow-up of digestive tract colonization

A total of 116 patients were analyzed because 9 patients were included in the ongoing study “FeDEX” registered at ClinicalTrials.gov (NCT03029078) to attempt the eradication of XDR colonization using FMT [12].

We observed a spontaneous decolonization in 48.2% (*n* = 56) of cases. Median time for decolonization was 49 days (1–1091). Among them 34% (*n* = 19) were decolonized within the first 30 days (D), 30.3% (*n* = 17) between D31-D90, and the remaining 35.7% (*n* = 20) after D90. All of them were followed-up with different time-points depending on the scheduled consultation or eventually re-admission. Their negative screens were confirmed with a median follow-up of 96 days (0–974). Two individuals exhibited re-colonization within the year.

Of note, 14.4% (*n* = 18) were followed after 1 year (of which 13 were decolonized and 5 colonized patients).

In order to identify factors related to the decolonization, we divided this cohort into 2 groups: decolonized and colonized patients. Results are summarized in Table 3. Our data achieved statistical significance for duration of hospitalization (*p* = 0.02) and duration of follow-up (*p* < 0.0001) which were longer for the decolonized patients. In the limit of the sample size in a subgroup analysis, we could not demonstrate any impact on decolonization between the different species, notably with an OXA-48 genotype (*p* = 0.35).

**Table 3:** Univariate analysis of factors which may have contributed to decolonization. Variables are compared between colonized and decolonized patients, using Fisher’s exact test

Variables	Colonized (*n* = 60)	Decolonized (*n* = 56)	*p*-value (α = 0.05)
Age, mean ± SD	61 ± 20.9	66 ± 18.4	0.17
Patients with a Charlson Comorbidity Index (CCI) < 5^a^, n (%)	35 (58.4)	28 (50.0)	0.46
Elapsed time in days to detect XDR consideration admission, median (min-max)	2 (− 420–101)	0 (−609–127)	0.34
Duration of hospitalization in days, median (min-max)	9 (1–273)	16.5 (2–290)	**0.02**
Duration of follow-up in days considering discharge, median (min-max)	7 (0–721)	99 (0–974)	**< 0.0001**
Antibiotic exposure prior to colonization, n (%)	21 (35.0)	25 (44.6)	0.34
Antibiotic exposure after being colonized, n (%)	12 (21.4)	5 (8.3)	*0.06*
Pooled duration of antibiotic regimen in days, mean ± SD	19.6 ± 15.3	21.1 ± 17.6	0.83
Occurrence of a sepsis due to XDR bacteria, n (%)	3 (5.0)	3 (5.4)	0.99
Carrying a CRE, n (%)	44 (73.3)	38 (67.9)	0.55
Harboring an OXA-48 CRE, n (%)	33 (55.0)	25 (44.7)	0.35
Returning back home, n (%)	43 (71.7)	44 (78.6)	0.52
Being transfer to another facility including rehabilitation, n (%)	7 (11.7)	9 (16.1)	0.59
Unfavorable outcome (death), n (%)	8 (13.3)	2 (3.6)	0.1

^a^Cohort was divided according to CCI median equal to 5. Patients with a CCI scores ≥5 are considered as severe and fragile

Bold data are significant and italicized is a trend to significant result

### Outcomes and future of patient care

The mortality rate was 8.8% (*n* = 11). Death was more likely attributable to underlying conditions than sepsis related to the XDR bacteria (*n* = 2).

Most of the patients (72.8%, *n* = 91) returned home, while 9.6% (*n* = 12) were transferred to rehabilitation, 8.8% were admitted to another acute healthcare facility and 3 patients were placed in palliative care.

### Patient care

We estimated that XDR carriage was associated with a larger care burden in 13.6% (*n* = 17) of cases.

- Prolongation of hospitalization (*n* = 8): Main reason was the absence of facility with the area of expertise in hygiene and enough paramedical staff to adhere to isolation precautions required for XDR organisms.
- Delayed surgery (*n* = 7): quite a few cases could not benefit from surgery to the lack of technical expertise on site and no direct possibility to arrange a medical transfer with the required precautions.

The rate was significantly lower in Kremlin-Bicêtre hospital in comparison to Raymond-Poincaré hospital, 2.9% (*n* = 2/70) versus 27.3% (*n* = 15/55), respectively (*p* < 0.0001). The median time for the delay was 32.5 days (15–300).

### Benefits from the cohorting

Of 1916 patients in contact with the XDR carriers before isolation, we screened 1603 patients. No secondary case was recorded, and thus no outbreak occurred in both sites.

We calculated that in the absence of dedicated units it would have required 26,730 screenings (198*3*(20 + 25)). Our estimation was based among 198 stays considering there are 25 beds for non-XDR carriers in Raymond-Poincaré and 20 beds in Kremlin-Bicêtre, and that each patient requires 3 negative swabs before stopping the screening procedures. Overall, both hospitals saved 25,127 swabs (26730-1603) and the equivalent number of agar plates during 2 years. The saving is estimated approximately around 25127*(2.5 + 0.7) = 80,406€ in our hospital group (AP-HP, Paris, France) without considering the labor costs.

## Discussion

In our present cohort, spontaneous clearance of XDR bacteria is not so rare and occurred in 48.2% of cases with a median time of 49 days and one-third of them were decolonized within a month. Those findings are concordant with the limited data available in the literature reporting natural clearance of MDRO and especially CRE [17–22] and VRE [23, 24] carriers during hospital settings. All of them are retrospective data, mainly concerning KPC cases occurring in Israel, except one prospective sub-study issued in China by Cheng et al. [22] which focused on NDM-1 isolates and reported a surprisingly high rate of 72.2% (*n* = 57) of decolonization within a short period (median delay of 30 days). Furthermore, sample size ranges from 66 to 125 patients and some studies present mixed population by adding patients in rehabilitation centers and long-term care facilities.

To our knowledge, this is the first study that describes natural decolonization of XDR (CRE and VRE together) carriers in an adult medical ward in Western Europe (France). Actually, in the literature only one study was conducted in Germany but concerned an outbreak of Carbapenem-Resistant *K. pneumoniae* related to a patient transferred from Greece [17] where the prevalence is deemed to be high (around 60% in 2013) [25]. Even if the prevalence of CRE is still rare in France in 2016 (< 1%) [25], considering the boarders we share with the Mediterranean countries, we should be wary of possible outbreaks.

Also, our cohort illustrates 2 interesting data. Firstly, decolonization does not seem to result from the type of microorganism (VRE versus CRE in Table 3). This is consistent with the review of the literature, showing a similar median of decolonization (around 6 months) between VRE [24] and CRE carriers [16]. Secondly, there is a trend of persistent bacterial carriage with XDR bacteria under antibiotic exposure (*p* = 0.06), in the limit of the sample size. Therefore, we could not detail the different antimicrobials and prescriptions duration impact on decolonization. Nevertheless, we can assume that ongoing antimicrobial therapy may slow down the clearance of XDR bacteria over time, considering antimicrobial agents impact the gut microbiota [26]. This hypothesis is concordant with Schechner et al. who argued that prior fluoroquinolones use was predictive of persistent CRE rectal carriage [21] and with Cheng et al. who reported an impact of the use of proton pump inhibitors in addition to antimicrobial therapy [22].

We also observed 2 patients retested positive for CRE after 2 negative swabs during follow-up, but both received antimicrobial agents during the time interval. Such observation reinforces our belief that there is a close link between antibiotic exposure and selective pressure on gut microbiota.

It is important to note that decolonized patients had longer LOS than colonized patients (*p* = 0.02). This may be explained by the fact that decolonized patients had also longer follow-up (*p* < 0.0001). Also, while patients are colonized, physicians are under pressure to expedite patient discharge to avoid in-hospital spread of XDR bacteria. Moreover, their follow-ups are difficult to undertake, because most of the patients returned back home (72.8%) mainly because of a refusal of other healthcare facilities to set up necessary precautions. Therefore, a majority of patients did not undergo screening after 1 year (85.6%).

Furthermore, we described differences along the continuum of patient care between the 2 main study sites (*p* < 0.0001). It can be explained by the fact that Kremlin-Bicêtre hospital has a dedicated rehabilitation unit for XDR carriers (up to 6 beds), isolated in contact precautions (for MDRO) which contributes to improve patient flow and also because Kremlin-Bicêtre hospital has bigger facilities with a larger technical expertise.

Moreover, there is a lack of a consensual definition of clearance, including VRE, partly due to heterogeneity in the studied populations [24]. In the present work, we considered at least 2 negative rectal swabs (> 1 week) as a baseline for the decolonization process while Lubbert et al. proposed a series of at least 4 consecutive negative rectal swabs (> 1 week) [17], which is one major point that limits the interest of all the above findings in real-life conditions. In 2017 in the 39 hospitals of AP-HP, the central committee for hygiene policy (CLIN) proposed a series of at least 3 negative swabs with one-year hindsight before considering the patient free from XDR bacteria. Yet in Garches, we reduce precautions from quarantine to contact precautions after 4 months of negative swabs, considering that patient might still excrete XDR bacteria as soon as he is exposed to antibiotics. Such long period of time can be sometimes really hard to face the patients, especially when they are not infected.

Therefore, we believe our findings might be of interest to guide hygiene policies accurately about reducing extra precautions on readmission to the healthcare system to lower a significant burden on healthcare provision. Besides, one solution to enhance bacterial clearance might be the FMT proposed by our team as a “fecal weapon” which showed promising results [14].

Finally, this study has several limitations to be considered. First, we experienced a high rate of people lost to follow-up after 1 year. This can be explained by the fact that once a patient has been discharged, it is very difficult to obtain a monthly rectal swab without evidence-based medicine. Second, we did not perform systematically PCR testing on follow-up swabs, considering that PCR was only legitimate for a rapid initial diagnosis of carriage and not indicated in routine surveillance considering its low positive predictive value of 16.6% [27] and would result in higher costs. Thirdly, whereas multiple hospitalizations (198 stays for 125 patients) were recorded in our analysis, we cannot warrant that some patients were hospitalized in other acute care facilities between the initial culture and the final follow-up.

## Conclusions

In a context of multidrug resistance development and a lack of new antimicrobial agents, a stringent surveillance of XDR bacteria is needed. Our study shows that a spontaneous decolonization occurred within the first 30 days in 16.4% of cases and up to 48.2% after day-90. This major point confirms that spontaneous decolonization is not so rare and does not seem to be static over time which is very encouraging. We observed a larger care burden in almost one-tenth of XDR carriers, because of their quarantine condition. This must be considered when physician decides to screen for a XDR bacteria. Finally, we can regret that a majority of patients did not undergo screening after 1 year, emphasizing the need for more monitoring and prospective studies.

## Abbreviations

CLIN: Central committee for hygiene policy
CRE: Carbapenem-resistant Enterobacteriacae
FMT: Fecal microbiota transplantation
ICU: Intensive care unit
IDS: Infectious disease specialist
LOS: Length of stay
MDRO: Multidrug-resistant organism
VRE: Vancomycin-resistant Enterococci
XDR: Extensively Drug-Resistant
